# Epidemiological Characteristics of Imported Influenza A (H1N1) Cases during the 2009 Pandemic in Korea

**DOI:** 10.4178/epih/e2012009

**Published:** 2012-12-31

**Authors:** Jun Kil Choi, Sang Won Lee, Bo Youl Choi

**Affiliations:** 1 Division of Quarantine Support, Korea Centers for Disease Control and Prevention, Cheongwon, Korea.; 2 Division of Epidemic Intelligence Service, Korea Centers for Disease Control and Prevention, Cheongwon, Korea.; 3 Department of Preventive Medicine, Hanyang University College of Medicine, Seoul, Korea.

**Keywords:** Pandemic influenza A (H1N1), Quarantine, Imported disease

## Abstract

**OBJECTIVES:**

Quarantine measure for prevention of epidemic disease and further evaluations of their efficiency are possible only by elaborating analyses of imported cases. The purpose of this study was to analyze descriptive epidemiological characteristics of pandemic influenza A (H1N1) cases imported to Korea.

**METHODS:**

We collected two sets of data. The first set, comprised daily reported cases of H1N1 obtained from local cities in accordance with government policy about mandatory reporting of all H1N1 cases during May 1 to August 19, 2009. The second set, including 372 confirmed imported H1N1 cases, identified from 13 National Quarantine Stations in the Korea Centers for Disease Control and Prevention from May 24 to December 31, 2009. However, given the lack of information on the nature of the imported H1N1 cases from the two data sets during the over lapping period from May 24 to August 19, we express the number of imported cases as a range for this period.

**RESULTS:**

We estimated that the number of imported H1N1 cases from May 1 to August 19, 2009, was between 1,098 and 1,291 and the total number of cases was 2,409 to 2,580. We found the number of imported cases was beginning to diminish as of August. A analysis of the second data set showed that the distribution of sex was similar (males 50.7%, females 49.3%) and the age distribution from 20 to 59 was 61.5% and that of 60 and over was 0.8% of the 372 cases. We identified 25 countries where people infected with H1N1 traveled and 67.5% were in Asia. But the proportion of cases (/1,000) by region shows Oceania (0.199), South America (0.118), Southeast Asia (0.071), North America (0.049), Europe (0.035), and Northeast Asia (0.016) in that order. The order of H1N1 peaking was the Southern Hemisphere, Tropics, and the Nothern Hemisphere.

**CONCLUSIONS:**

This study provided information that could make possible the evaluation of the government quarantine measure for stopping imported disease from causing community-acquired spread in the future.

## INTRODUCTION

The number of overseas travelers to Korea has increased since the overseas travel was liberalized in 1989. About 9,494,000 people traveled to foreign countries in 2009 [1]. It is worth noting that airplanes carried more than one billion travelers worldwide [2,3], who have been the main carriers of infectious diseases from foreign countries each year [4]. It was confirmed that infectious diseases could be spread by airplanes through the severe acute respiratory syndrome (SARS) pandemic, which infected about 8,000 people in 30 countries and took about 700 people's lives in Hong Kong in 2003 [5]. To minimize the domestic spread of infectious diseases from foreign counties, 13 national quarantine stations identify areas affected by infectious diseases. Travelers who have visited these areas are subjected to quarantine including fever observation, health questionnaire including items on personal travel information and symptoms, the isolation of suspected patients, and laboratory diagnoses. The quarantine system is known to the public for its strict border control measure that enforced the containment (quarantine and isolation) during the pandemic period for influenza A (H1N1) in 2009. And the system has been more important for preventing disease epidemics. However, there has been no domestic analysis of characteristics of the cases of quarantine and imported disease. Although the government took steps to prevent severe acute respiratory syndrome from entering the country in 2003, a general evaluation of the government's responses was performed only after the H1N1 pandemic in 2009 [6,7]. Meanwhile, epidemiological analyses of H1N1 cases had already been performed during H1N1 epidemic periods in some countries, such as Singapore [8]. In addition, studies evaluating the effectiveness of quarantine have been reported recently [9,10]. Quarantine measure for the prevention of epidemic diseases and further evaluations of their efficiency are possible only by elaborate analyses of imported cases. Thus, this study investigated the descriptive epidemiological characteristics of H1N1 cases in Korea during 2009 pandemic period by reviewing the daily H1N1 occurrence and imported H1N1 cases identified by the quarantine process of 13 National Quarantine Stations to provide basic data for improving the effectiveness of quarantine measure.

## MATERIALS AND METHODS

**Figure 1:**
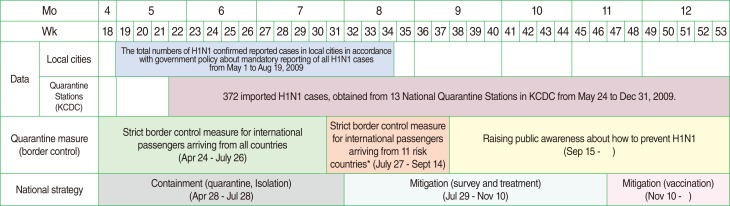
Two data sources and quarantine (national strategy) measures for H1N1 by week and month in 2009. H1N1, influenza A; KCDC, Korean Centers for Disease Control and Prevention. ^*^Eleven high risk countries had current epidemics: the United States, Canada, Mexico, United Kingdom, Spain, Australia, New Zealand, Chile, Thailand, Hong Kong, and the Philippines.

The subjects of this study were all confirmed H1N1 patients (The daily reported cases from local cities) and only imported H1N1 patients (incoming travelers) confirmed by 13 National Quarantine Stations (Figure 1). The daily reports on the patients who were confirmed by polymerase chain reaction (PCR) tests in all of local cities were collected from May 1 to August 19, 2009, The occurrence dates, cases imported and community-acquired infections, and infection routes of those cases were investigated. Imported H1N1 cases were defined as patients who had overseas travel history within the previous 7 days. Community-acquired H1N1 cases were defined as patients who were surmised to have had contact with other patients and had not traveled overseas. The other subject group included 372 imported H1N1 patients who were confirmed by PCR tests through 13 National Quarantine Stations in the Korea Centers for Disease Control and Prevention (KCDC) from May 24 to December 31, 2009. The information sources were from the Health Questionnaire including personal travel information. Weekly epidemiological analyses were performed from the 22nd week to 53nd week. Descriptive analyses of data from the Health Questionnaire including sex, age, entry dates, and countries visited were performed by SPSS version 12.0. (SPSS Inc., Chicago, IL, USA). However, the number of imported H1N1 cases was treated as a range with a minimum and maximum from May 24 to August 19, 2009 because any overlap of the cases from the reported data and quarantine data could not be confirmed.

## RESULTS

### Trends in the occurrence of total imported influenza A (H1N1) cases

**Figure 2:**
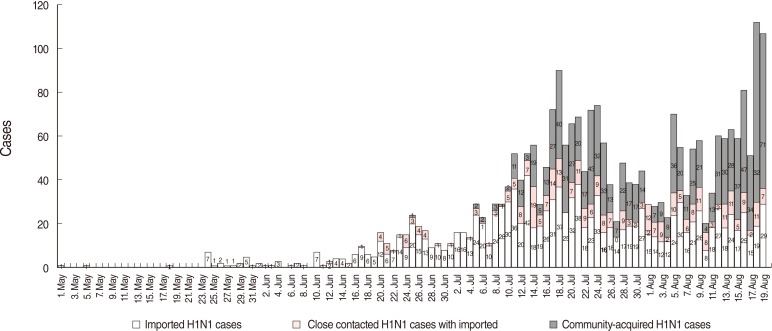
Infection sources reported at 13 National Quarantine Stations in KCDC and local cities until Aug 19. KCDC, Korea Centers for Disease Control and Prevention.

From the data collected by local cities from May 1 to August 19, 2009, it was estimated that the number of imported H1N1 cases was from a minimum of 1,098 to a maximum of 1,291, and the total number of H1N1 cases was at least 2,409 and a maximum of 2,580. Reviewing the trend for the early outbreak of H1N1, the first imported H1N1 case was identified on May 2, 2009, and 2 additional cases were found by tracing the infection route and the contacts of the first case [11]. What was assumed to be the first community-acquired H1N1 case was reported on June 25. In reviewing monthly data by infection route, the total number of H1N1 cases was 2,580 with the maximum estimate as follows: 24 cases including 22 imported cases, and two putative cases by close contact were reported in May, 212 cases in June, 1,318 cases in July, and 1,026 from August 1 to 19. The percentages of imported H1N1 cases were estimated to be 91.7% (22 cases) in May, 84.9% (180 cases) in June, 54.6% (719 cases) in July, and 36.1% (370 cases) from August 1 to 19. In other words, beginning in August, the number of putative community-acquired H1N1 cases had exceeded that of imported cases, and community-acquired H1N1 had spread all over the country (Figure 2).

### Epidemiological characteristics of H1N1 cases from the quarantine

#### Demographic characteristics

The distributions by sex, age, and country of 372 H1N1 patients who were confirmed by 13 National Quarantine Stations only were investigated for 32 weeks, from May 24 to December 31.

The number of male patients was 186 and that of female patients was 181 out of 367 patients, which accounts for all but 5 who omitted their sex from the health questionnaire. The main age distribution was from 20 to 29 years old (37.9%). The number of patients 60 years old and above was 3, comprising 0.8% of the total. Reviewing 367 patients, that is, all but 41 who did not fill out their occupation information in the health questionnaire, the number of civilians was 107 (29.1%), that of elementary, middle, and high school students was 103 (28.1%), that of undergraduate and graduate students was 89 (24.2%), and that of preschoolers was 27 (7.4%) (Table 1).

Among 367 patients, that is, all but 8 who did not fill out their nationality information, the number of Koreans was 328 (89.4%) and the number of non-Koreans was 31 (8.4%).

#### The status of countries visited

The number of the countries visited by the 361 H1N1 patients, that is, all except 11 who did not report the countries they visited on the health questionnaire, was 25.

The country with the highest ratio of H1N1 cases per 1,000 entrants among the countries visited was Guam, followed by Hong Kong, New Zealand, Australia, and the Philippines, in that order. The region with the highest ratio of H1N1 cases was Oceania, followed by South America, Southeast Asia, North America, Europe, and Northeast Asia in that order (Table 2).

#### The status of entry for H1N1 patients

**Figure 3:**
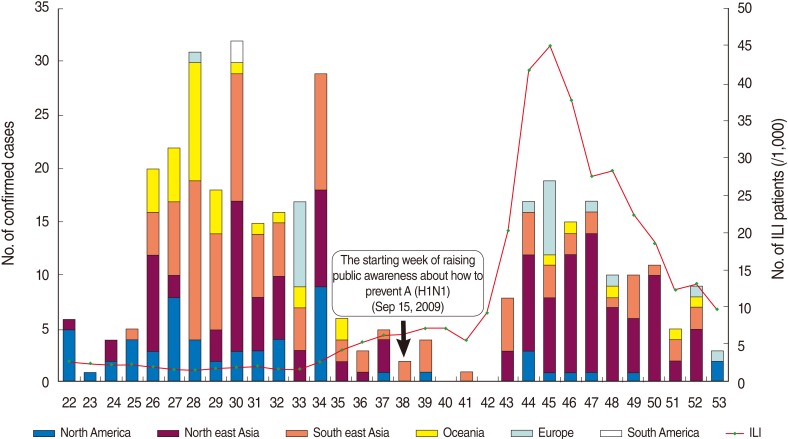
The composition of the inflow of confirmed imported cases by region and week and the status of influenza like illness (ILI) in Korea.

The highest inflow of confirmed imported cases by region and by week was from North America and Northeast Asia, followed by Southeast Asia, Oceania, Europe, and South America, in that order (Figure 3). In a review by country, H1N1 cases were found first in travelers from the United States and Japan in the 22nd week. H1N1 cases were found in travelers from Hong Kong during the 24th week; from the Philippines during the 25th week; from Australia in the 26th week; from New Zealand, Canada, and Thailand during the 27th week; and from Brazil in the 30th week. The ratio of influenza-like-illness (ILI) in Korea had rapidly increased from the 41st week and reached its peak, 44.96 cases out of 1,000, at the 44th week. This corresponded with the H1N1 trend of quarantine numbers during the winter (Figure 3).

## DISCUSSION

**Figure 4:**
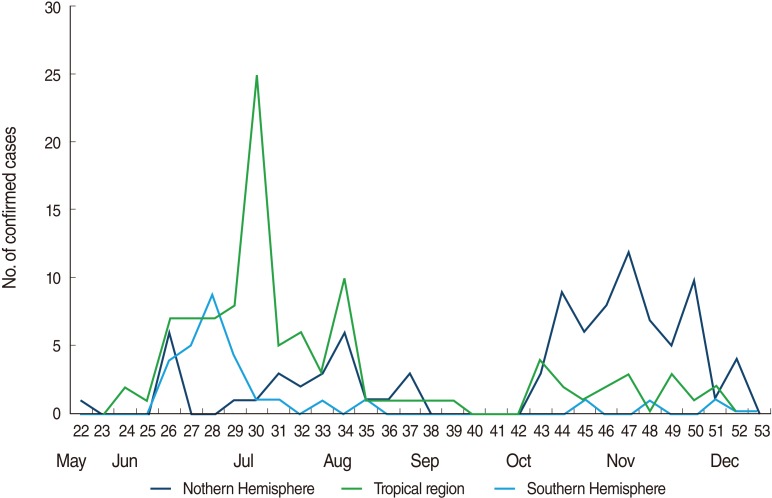
Epidemic curves of confirmed imported cases of influenza A (H1N1) in 2009 detected from 13 National Quarantine Stations by geographic and climate area.

In the middle of March 2009, an increase in nonspecific ILI was observed by Mexico's Ministry of Health. Two H1N1 cases were reported in California by the United States Centers for Disease Control and Prevention (US CDC) in the middle of April 2009. By the end of the third week in April, it was confirmed that patients who had influenza or pneumonia symptoms were infected by the same H1N1 virus in Texas and Mexico [12]. The World Health Organization (WHO) announced that this virus was a different type than the previous ones found in pigs and humans [13]. According to this announcement, influenza A prevention systems were activated all over the world. In Korea, the containment (quarantine and isolation) level of the quarantine masure (border control) was taken to prevent the inflow of H1N1. During this period, fever tests and health questionnaire collection were performed by 13 National Quarantine Stations on every entrant from all countries. Beginning July 27, 2009, the range of travelers subject to quarantine was reduced to travelers from 11 high risk countries. After September 15, border measeure was converted gain to the level of raising public awareness about how to prevent H1N1. As confirmed H1N1 cases had been reported in most countries, and the number of patients had increased, the WHO no longer collected the number of confirmed patients beginning July 17, 2009 [14]. However, local cities had reported daily data in accordance with government policy about mandatory reporting for suspected imported and community-acquired H1N1 patients beginning May 1 and continued to do so through August 19, 2009. Reviewing the daily trend of H1N1 cases in Korea, the first imported case was confirmed on May 2, 2009. On June 25, the first putative community-acquired case was reported. The gap between the official date, July 11, and the date reported by local cities and provinces of the first putative community-acquired case was about 2 weeks because the data from local cities and provinces was reported daily; however, the official data was reported after additional epidemiological investigation. The number of putative community-acquired H1N1 cases exceeded that of imported cases, and community-acquired H1N1 had spread all over the country since August. In China, which has the same seasonal conditions as Korea, the number of putative community-acquired H1N1 cases exceeded that of imported cases on July 28, 2009 [15], with a trend similar to that of Korea. Meanwhile, in Japan, public health actions taken included the closing of schools and the delay of public assemblies because the epidemic had already spread, with 321 H1N1 patients reported by May 23, 2009 [6]. Japan reduced its quarantine measure from the isolation level to the mitigation level on May 21 which was five days later after confirming the first community-acquired H1N1 case on May 16, 2009 [16]. However, Korea and China maintained the isolation level by the end of July and July 10, respectively. The ratio of H1N1 cases per 100,000 people in Japan was three times higher, at 0.29 patients, than the ratio in Korea, with 0.09 patients at the end of May 2009. However, by August 9, 2009, the ratio of H1N1 cases per 100,000 people was lower in Japan (3.89 cases) than Korea (4.02 cases). It should be noted that unlike Japan's focus on treatment instead of diagnosis of H1N1 early in the epidemic, the use of active PCR tests could have increased the official H1N1 ratio in Korea. The next part of the investigation concerned the trend of 372 imported H1N1 patients who were confirmed by 13 National Quarantine Stations from the 22nd week, 2009, to the 53rd week. The average number of imported H1N1 cases per week identified by quarantine was 11.6 for those 32 weeks in 2009. The average number of weekly imported H1N1 cases during the period of the containment (quarantine and isolation) level was 15.6 for 9 weeks, and during the period of the mitigation level, it was 13 cases for 7 weeks. The average number for imported H1N1 cases per week during the period at the level of raising public awareness was 8.7 for 16 weeks. The decrease in the countries visited that triggered quarantine and the change of quarantine measure could have played a role in the decrease in the number of H1N1 cases per month. In other words, the total number of entrants coming from foreign countries had been subject to quarantine during the period with the containment (Quarantine and Isolation) level, but the number of subject to quarantine was reduced to only entrants from countries classified as high risk countries during the period at the mitigation level. In addition, during the period of the level of raising public awareness, a prompt visit to the doctor's office was recommended to those with symptoms rather than tests for entrants. In the assessment of the epidemiological characteristics of imported H1N1 cases, it was found that the distribution by sex was similar, 50.7% of the cases were male and 49.3% were female. The adults aged 20 to 59 years old comprised 61.5%, and the ones 60 years old and above comprised 0.8%. The US CDC reported that the cross-reaction antibody for H1N1 did not exist in children; however, among adults 18 to 64 years old, 6-9% had the antibody for H1N1, and 33% of those 65 years old and above had it [17]. Of all of the H1N1 patients, 75.6% were younger than 30 years old. This is similar to the European data, in which the percentage of H1N1 patients younger than 30 years old was 80% [18]. Among 367 patients, that is, all except 8 who did not report their nationality, 328 (89.4%)were Korean citizens and 31 (8.4%) were non-Koreans. Thus, the vast majority of the cases were from Korean travelers returning from overseas. Of the total cases, the groups including the primary, middle school, high school, undergraduate, and graduate students comprised 52.3% (Table 1). Notably, during summer vacation, which was from the middle of July to the end of August, cases from these groups were 32% of the total cases. Travelers from foreign countries are the main carriers for pandemics [4]. In addition, the ratio of student travelers is high. This implies that thorough control and the education of student travelers is necessary. When the data on countries visited are considered, it can be seen that 67.3% of the total cases were travelers from elsewhere in Asia. However, the highest ratio of H1N1 patients per 1,000 people by region was 0.199 in travelers from Oceania, followed by South America with 0.118, Southeast Asia with 0.071, North America with 0.049, Europe with 0.035, and Northeast Asia with 0.016, in that order. The areas which patients had visited were classified into three parts, which were the Northern Hemisphere, the Tropics, and the Southern Hemisphere, because the trends of seasonal epidemic influenza were different by the area [19]. The number of cases from the Northern Hemisphere including China and Japan was 93 out of 372, from tropical regions including Hong Kong, Malaysia, the Philippine, Singapore, and Vietnam was 104, and from the Southern Hemisphere including Australia and New Zealand was 29. By these data, the comparisons of the ILI ratio for the Northern Hemisphere, the Tropics, and the Southern Hemisphere in the Western Pacific Region were performed [20]. Comparing the graphs of these data, the monthly data for H1N1 were similar, and the order of peaking was the Southern Hemisphere, Tropics, and the Northern Hemisphere (Figure 4). Reviewing the H1N1 data in the 30th weeks, which had the highest number of cases, there were 7 cases from the Philippines, 13 cases from Singapore, 5 cases from Vietnam, and no cases from Hong Kong or Malaysia. The comparison between seasonal influenza by region and the trends of the H1N1 outbreak is useful in developing effective control strategies for future pandemics [21]. In case of the Southern Hemisphere, although there are geographical and demographical differences, the H1N1 pandemic showed highly consistent patterns in 2009 [22]. This geographical and epidemiological information suggests countries that should be priorities for strict border control measure when new pandemics occur. In other words, this data would suggest effective selected and focused quarantine measure given limited manpower. This study has the limitation of uncertainty about the nature of the overlap of imported H1N1 cases between local citis data and 13 National Quarantine Stations data from May 24 to August 19, 2009. Thus, the number of imported H1N1 cases was expressed as a range with a minimum and maximum during that period. Nevertheless, this study is meaningful in that it provides the basic data for developing more effective quarantine measure because it is the initial attempt to analyze descriptive epidemiological characteristics of H1N1 cases imported to Korea who were confirmed by quarantine and daily report data from local cities for a certain period.

## Figures and Tables

**Table 1:**
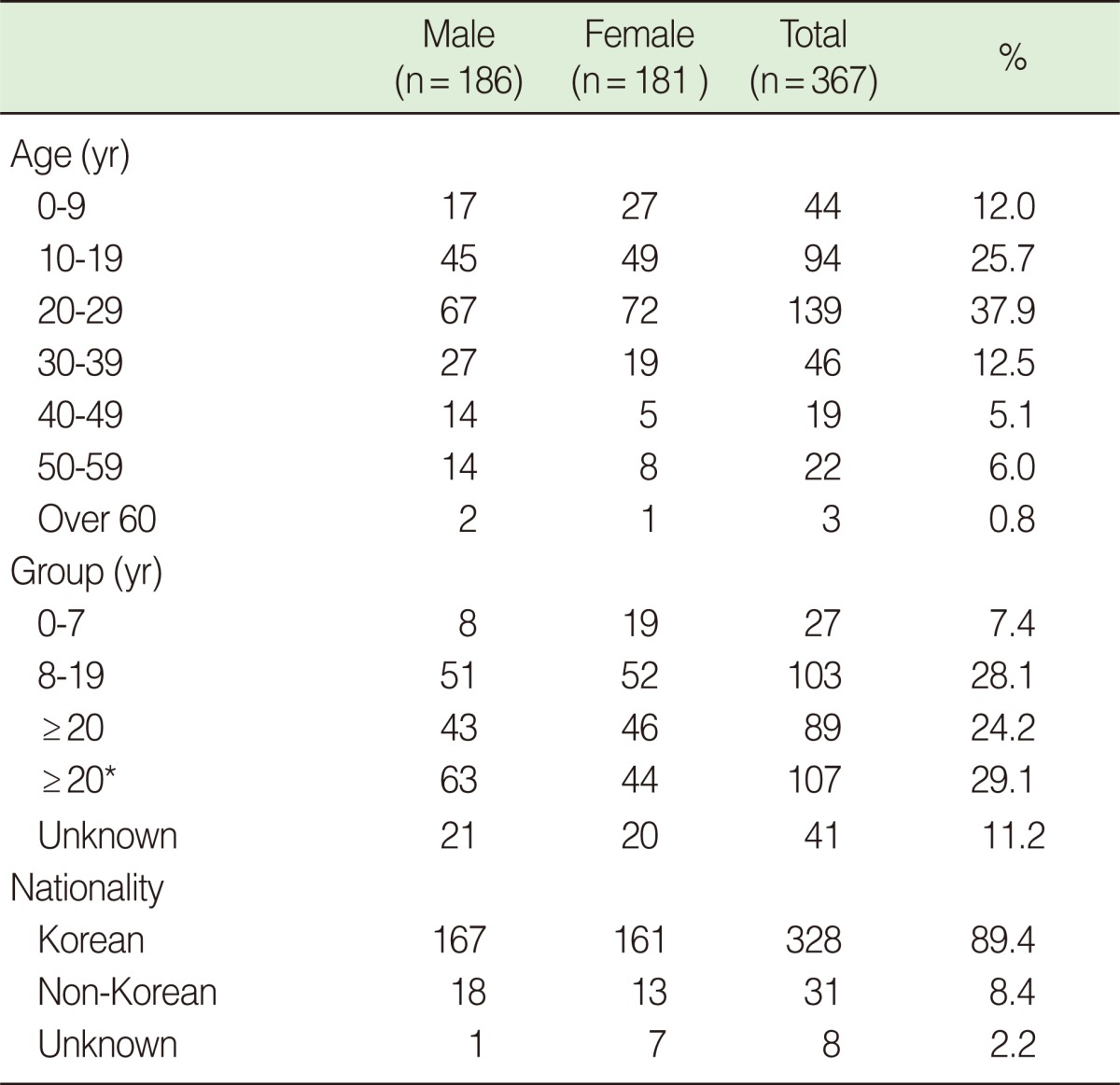
Composition of imported confirmed cases (from May 24 to Dec 31, 2009)

^*^Not fill out their occupation information as students.

**Table 2:**
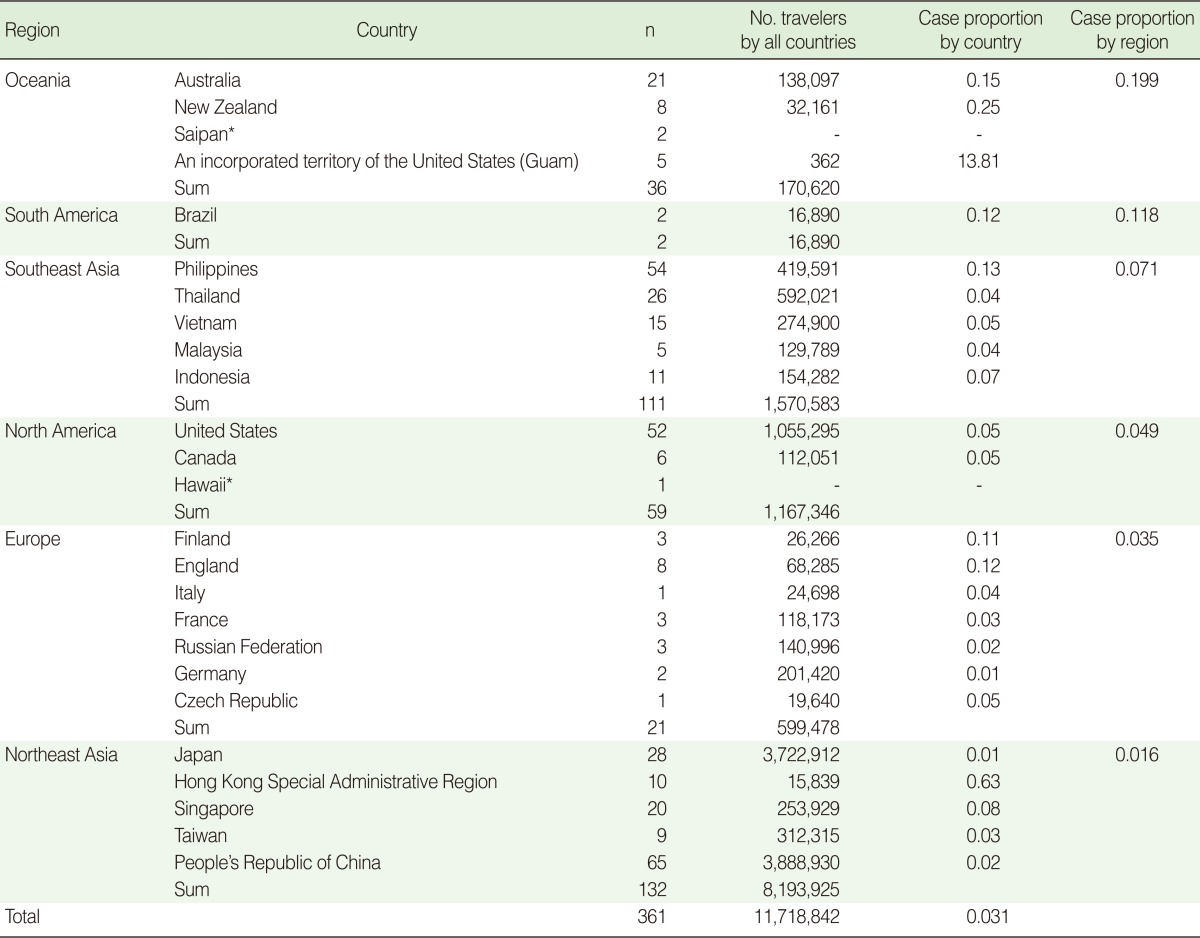
The composition of the inflow of confirmed imported cases by country and by region (from May 24 to Dec 31, 2009)

^*^Data missing for the number of entrants.
